# The Role of *Wolfiporia cocos* (F. A. *Wolf*) Ryvarden and Gilb. Polysaccharides in Regulating the Gut Microbiota and Its Health Benefits

**DOI:** 10.3390/molecules30061193

**Published:** 2025-03-07

**Authors:** Yong Lai, Xin Lan, Zhicheng Chen, Guanhua Lou, Ying Li, Chang Liu, Jianan Feng, Xi Li, Yu Wang

**Affiliations:** 1 Institute of Traditional Chinese Medicine of Sichuan Academy of Chinese Medicine Sciences, Chengdu 610031, China; yonglai3210@163.com (Y.L.); louguanhua@sc2zzy.com (G.L.); liying@sc2zzy.com (Y.L.); liuchang@sc2zzy.com (C.L.); fengjianan@sc2zzy.com (J.F.); 2 School of Basic Medical Sciences, Southwest Medical University, Luzhou 646000, China; lanxin202202@163.com; 3 School of Clinic Medical Sciences, Southwest Medical University, Luzhou 646000, China; 15281207768@163.com

**Keywords:** *Wolfiporia cocos*, polysaccharides, gut microbiota, pharmacological function, health benefits

## Abstract

*Wolfiporia cocos* (F. A. *Wolf*) Ryvarden and Gilb. is a widely used herb in China, belonging to the large fungi of the family Polyporaceae. *P. cocos*; it consists of a variety of biologically active ingredients such as polysaccharides, triterpenes, and sterols, and is considered a treasure in traditional Chinese medicine (TCM). Notably, *P. cocos* polysaccharides, as the most prominent constituent, are of interest for their superior anti-obesity, anti-tumor, anti-inflammatory, antioxidant, and immunomodulatory activities. *P. cocos* polysaccharides can be divided into water-soluble polysaccharides and water-insoluble polysaccharides, which may contribute to their diverse biological functions. Numerous scholars have focused on the extraction process, structural identification, and classical pharmacological pathways of *P. cocos* polysaccharides, but there are few systematic reviews on *P. cocos* polysaccharides regulating the gut microbiota. Natural products and their active ingredients are closely related to intestinal health, and further exploration of these mechanisms is warranted. This review summarizes the recent cases of *P. cocos* polysaccharides regulating the gut microbiota to promote health and discusses their relationship with bioactive functions. It aims to provide a basis for exploring the new mechanisms of *P. cocos* polysaccharides in promoting intestinal health and offers a new vision for the further development of functional products.

## 1. Introduction

*Wolfiporia cocos* (F. A. *Wolf*) Ryvarden and Gilb. is a common and famous traditional Chinese medicine, mainly distributed in China, America, Oceania, Japan, and Southeast Asia [1]. *P. cocos* typically parasitizes the roots of pine trees and is classified as the dried mycelium of the fungus *Poria cocos* (Schw.) *Wolf*, belonging to the family Polyporaceae [2]. It is a kind of a large fungal plant, which is popular and has unique medicinal and edible values [3]. As one of the Chinese treasures in the past and present, *P. cocos* has a great demand in the market and has broad development prospects.

*P. cocos* has been used as a traditional Chinese medicine and dietary supplement for over 2000 years, and it has been described as a “medicine for all seasons”. According to TCM, *P. cocos* has the effects of inducing diuresis and seepage of dampness, strengthening the spleen, and tranquilizing the heart [4]. Modern pharmacological studies have indicated that *P. cocos* has a variety of biological activities such as hypoglycemic, hypolipidemic, anti-tumor, anti-inflammatory, antioxidant, and immunomodulatory effects [5,6]. The active ingredients of *P. cocos* include polysaccharides, triterpenes, sterols, amino acids, proteins, nucleotides, and trace elements [7]. Polysaccharides are a class of macromolecular carbohydrates, and polysaccharides are the most important active ingredient in *P. cocos*, accounting for 70–90% of the total mass of the fungus [8]. *P. cocos* triterpenoids are diversified and have significant activities, mainly including *P. cocos* acid, tulmoic acid, and ibruric acid [9]. *P. cocos* polysaccharides and triterpenes are widely used and rich in pharmacological activities, mainly in the fields of anti-tumor, immunity regulation, and hypoglycemia effects [10]. In recent years, isolation technology and pharmacological research on the active ingredients of *P. cocos* have developed rapidly [11]. However, the active ingredients in *P. cocos* have not been fully clarified, and the biological activities of some components are still unknown. In addition, the pharmacological mechanisms and targets of action of *P. cocos* have not been fully elucidated, especially the mechanism of its multicomponent synergistic action. Therefore, the scientific research community needs to adopt more techniques and methods to carry out more in-depth studies and to reveal the mechanism of action of *P. cocos* ingredients to improve diseases, so as to better utilize its edible and medicinal value.

The gut microbiota is a complex ecosystem composed of numerous microorganisms in the gastrointestinal tract, and its composition and diversity play a key role in maintaining the health of the body [12,13]. Imbalances in the gut microbiota contribute to the development and progression of diseases including obesity, diabetes, antibiotic-associated diarrhea (AAD), inflammatory bowel disease (IBD), chronic liver disease, and colorectal cancer [14,15]. Numerous studies have shown that natural products derived from TCM can play a therapeutic role by remodeling the intestinal microecological structure, modulating flora-related metabolites, and influencing intestinal absorption and transport [16,17]. Targeting the gut microbiota for health promotion has great potential, and it is important to explore the specific mechanisms by which natural products modulate the gut microbiota.

Numerous scholars have studied the extraction process, structural identification, and classical pharmacological pathways of polysaccharides, but there are few systematic reviews on *P. cocos* polysaccharides regulating the gut microbiota [18]. Therefore, this paper reviews the bioactive activities and health benefits of *P. cocos* and focuses on the potential relationship between PCPs and the gut microbiota. This review aims to provide a basis for the mechanisms by which *P. cocos* and its active ingredients modulate the gut microbiota to ameliorate diseases, and to provide a new strategy for further research on the potential development of PCP bio-actives in food and drugs.

## 2. Polysaccharides, the Main Component of *P. cocos*

### 2.1. P. cocos Polysaccharides and Other Effective Active Ingredients

The bioactive ingredients in *P. cocos* include polysaccharides, triterpenes, sterols, volatile oils, proteins, amino acids, nucleotides, and trace elements [19]. Modern pharmacological studies have revealed that polysaccharides are the predominant pharmacologically active ingredients found in *P. cocos*, accounting for about 70% to 90% of the total weight of the whole fungus [20]. Polysaccharides are divided into heteropolysaccharides and homopolysaccharides according to their monosaccharide composition [21]. The polysaccharides in *P. cocos* are mainly divided into a class of branched-chain β-(1–3)-d-glucan containing (1–6) and (1–2) and another class of heteropolysaccharides composed of glucose, arabinose, fucose, mannose, xylose, galactose, and other monosaccharides [22]. Despite continuous advancements in polysaccharide identification techniques, current methods still face limitations in accuracy, consistency, and a comprehensive consideration of dynamic changes in microbial communities, as well as a lack of standardized protocols. These issues may hinder the in-depth understanding of the structure–function relationships of polysaccharides and limit their applications in microbiota research. Triterpenoids are mainly found in *P. cocos* mushroom kernel and skin, containing acid, alcohol, protein, and other components with antioxidant, anti-tumor, anti-inflammatory, and many other pharmacological effects [23]. *P. cocos* polysaccharides show excellent immune response, antioxidant, and anti-tumor activities, and triterpenoids exert their anti-tumor effects by modulating cell signaling and inhibiting tumor cells [24]. It has been shown that lasagna triterpenoids in *P. cocos* extract enhance nonspecific immunity by activating natural killer cells (NK), promoting interferon gamma (IFN-γ) secretion by type 1 helper T cells (Th1), and inhibiting the production of IL-4 and IL-5 [25]. In addition, Poria Acid (PA), another triterpenoid saponin, reduced the invasion and metastasis of gastric cancer cells by inhibiting the EMT process and the expression of MMP proteins [26]. Additionally, other active ingredients in *P. cocos*, such as sterols and ergosterol, have immunomodulatory effects, and alpha-cedrol in volatile oils has anti-inflammatory effects [26].

### 2.2. Extraction and Structural Characterization of P. cocos Polysaccharides

PCPs were mostly extracted by hot water extraction, alkali extraction, enzyme extraction, supercritical fluid extraction, and microwave-assisted and ultrasound-assisted extraction [27]. These methods exhibit distinct trade-offs: hot water and alkali extractions are simple and cost-effective but may degrade polysaccharide structures under harsh thermal or alkaline conditions; enzyme extraction offers mild specificity yet faces limitations in cost and scalability; supercritical fluid extraction is environmentally benign but equipment-intensive; while microwave and ultrasound techniques enhance yield and speed at the risk of localized overheating or radical-induced bioactivity loss. Evidence has demonstrated that deep eutectic solvent (DES) extraction for polysaccharide extraction has the advantages of high efficiency and high yield [28]. A study found that the DES method using a solid–liquid ratio of 1:10 and a ratio of choline chloride to oxalic acid of 1:2 gave a polysaccharide yield of 33.67% after 45 min of extraction [29]. Additionally, Guo et al. also found that the DES method using ternary butylene glycol with a 300 mesh particle size and a solid–liquid ratio of 30 mL/g gave a polysaccharide yield of 55.02% after 30 min of extraction [30]. With the development of instrumentation, as well as extraction, separation, purification, and structure identification techniques, the study of polysaccharides has advanced rapidly [25]. The current methods for polysaccharide extraction and purification are often limited by low yields and insufficient purity, which may introduce contaminants and affect the accuracy of their biological effects on microbiota. To address these issues, there is a need for standardized protocols to improve the yield and purity of polysaccharide preparations, thereby ensuring reliable and reproducible results in microbiota research. The analytical detection of *P. cocos* polysaccharides mainly includes the molecular weight of polysaccharides, monosaccharide composition, and glycosidic bond type [31]. This information can be identified with increasing accuracy by advanced physicochemical methods, chromatographic techniques, and spectroscopic analysis [32]. *P. cocos* polysaccharides can be divided into water-soluble polysaccharides and alkali-soluble polysaccharides according to the different extraction methods [33]. Water-soluble polysaccharides in *P. cocos* have a low content but have significant biological activities, such as anti-tumor and anti-inflammatory effects [34]. Compared with water-soluble polysaccharides, alkali-soluble polysaccharides have a higher content in *P. cocos*, which can reach 93%; however, these polysaccharides have poor water solubility and relatively low bioactivity, and especially almost no anti-tumor activity [35].

### 2.3. Chemical Modification of P. cocos Polysaccharides

Chemical modification is a widely used modification method employed to change the physicochemical properties and biological activities of polysaccharides by introducing different substituents such as carboxyl, methyl, sulfate, and hydroxyl groups into the polysaccharide molecules [36]. Structural modification of *P. cocos* polysaccharides not only improves the water solubility and stability of polysaccharides but also enhances their biological activities, such as anticancer, antioxidant, anti-inflammatory, and immunomodulatory effects [37]. In addition, chemical modification can help to improve the pharmacokinetic properties and bioavailability of polysaccharides, thus increasing their potential as drugs or functional foods. However, common modification methods, such as acetylation and sulfation, often involve toxic reagents and may generate by-products that could compromise the purity and safety of the modified polysaccharides. Therefore, while chemical modification improves the functionality of polysaccharides, it is essential to optimize the modification processes to minimize the introduction of toxic substances and impurities, ensuring their safe application in food and pharmaceutical industries [38]. It has been shown that by introducing carboxymethyl (-CH_2_COOH) into the polysaccharide molecule, the solubility and adsorption capacity of the polysaccharide, as well as its anti-tumor and antioxidant activities, can be increased, [39]. Sulphation is the introduction of sulfate (-SO_4_^2−^) into the polysaccharide molecule, which usually increases the water solubility and anticoagulant activity of the polysaccharide while potentially enhancing its anti-tumor and antiviral activities [40]. Therefore, in order to improve the water solubility and biological activity of *P. cocos* alkali-soluble polysaccharides, they are usually chemically modified by carboxymethylation and sulfate esterification [41]. For example, carboxymethyl porin polysaccharide (CMP) showed good anti-tumor effects, exerting its effects by modulating NF-κB, Nrf2-ARE, and the mitogen-activated protein kinase/P38 protein kinase/c-Jun amino-terminal kinase pathway [42].

**Figure 1:**
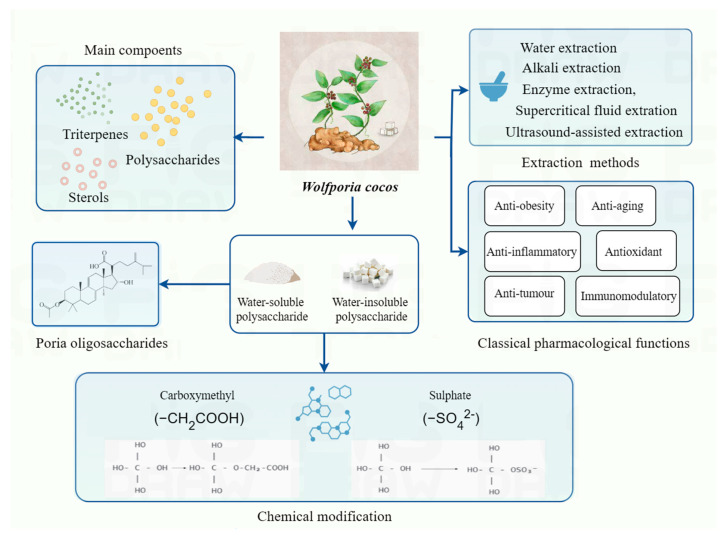
The main active ingredients of *P. cocos* are polysaccharides, triterpenes, and sterols. The extraction and purification methods of PCPs include hot water extraction, alkali extraction, enzyme extraction, supercritical fluid extraction, microwave-assisted and ultrasound-assisted extraction, and deep eutectic solvents (DESs) extraction. The pharmacological functions of polysaccharides include anti-inflammatory, anti-obesity, anticancer, and immunomodulatory functions. PCPs are classified into water-soluble polysaccharides and water-insoluble polysaccharides, and water-insoluble polysaccharides with poor water solubility are β-(1–3)-d-glucan, which are usually modified by sulfate esterification and carboxymethylation.

Notably, *P. cocos* oligosaccharides (PCOs) are a class of polymers and low-molecular-weight polysaccharides prepared by enzymatic degradation, containing sugar the residues of (1–2)-β-D-Glc_p, (1–2)-α-D-Glc_p, and (1–4)-α-D-Glc_p [43]. PCOs exhibit a wide range of applications in the pharmaceutical and food fields, with a variety of biological activities, including anti-tumor, antiviral, anti-inflammatory, immunomodulatory, and antioxidant effects [24]. Furthermore, they can improve disorders of glycolipid metabolism and protect the structural and functional integrity of the intestinal barrier by regulating intestinal flora [44]. Compared to *P. cocos* polysaccharides, PCOs have better water solubility and bioavailability due to their lower molecular weight, which makes them exhibit stronger antioxidant, immunomodulatory, and anti-tumor properties. To sum up, oligosaccharide degradation and the chemical modification of *P. cocos* polysaccharides, such as carboxymethylation, sulfation, and phosphorylation, can improve their water solubility, increase their molecular flexibility and stretchability, and thus enhance their biological activities, including anti-tumor, immunomodulatory, antioxidant, and anti-inflammatory effects, which makes the modified polysaccharides have a wider range of applications in the field of pharmaceuticals and functional foods (Figure 1).

## 3. Bioactive Functions of *P. cocos* Polysaccharides

Polysaccharides have great potential to be developed into functional prebiotics and therapeutic drugs due to their wide range of biological activities, making the study of their pharmacological activities and mechanisms a key research area [45]. For the past few years, plenty of studies have revealed the role and mechanism of applying *P. cocos* polysaccharides in the treatment of various diseases, and its pharmacological activities are mainly focused on anti-inflammatory, anti-obesity, anti-tumor, and immunomodulatory functions [46,47] (Table 1).

### 3.1. Anti-Obesity Effects of P. cocos Polysaccharides

*P. cocos* polysaccharides inhibit high-fat-diet-induced metabolic disorders by regulating host metabolic pathways [61]. Polysaccharides can change the synthesis and composition of bile acids, affecting the digestion and absorption of lipids, and then promote the oxidative utilization of fatty acids and reduce the accumulation of fat in the liver, which in turn exerts anti-obesity effects [62]. Meanwhile, *P. cocos* polysaccharides can improve the symptoms of diabetic patients by increasing glucose tolerance, improving abnormal glucose tolerance, and improving glucose metabolism disorder [63]. It was found that 95% pure *P. cocos* polysaccharide (PCP) intervened in atherosclerosis induced by a high-fat diet by inhibiting the activation of the TLR4/NF-κB pathway in the aorta and lowering inflammatory factors and lipid levels [48]. Wang et al. found that PCP reduced serum and liver lipid levels; affected fatty acid metabolism, bile acid metabolism, and metabolic pathways of the tricarboxylic acid cycle; and inhibited lipid synthesis and uptake to improve NAFLD [49]. Zhu et al. showed that PCO can significantly inhibit lipid metabolism disorders and reduce lipid accumulation and inflammation in blood and liver tissues to effectively ameliorate lipid metabolism disorders induced by a high-fat diet [50].

In summary, *P. cocos* polysaccharides exert anti-obesity effects and ameliorate high-fat-diet-induced metabolic disorders by modulating host metabolic pathways, altering bile acid composition to influence lipid digestion and absorption, promoting fatty acid oxidation and utilization, reducing hepatic fat accumulation, increasing glucose tolerance, improving glucose metabolism, inhibiting activation of the TLR4/NF-κB pathway in atherosclerosis, and decreasing inflammation and lipid-level disorders.

### 3.2. Anti-Inflammatory Activity of P. cocos Polysaccharides

*P. cocos* polysaccharides can reduce inflammatory response and the release of inflammatory factors [43]. Numerous studies have shown that *P. cocos* exerts anti-inflammatory effects by inhibiting the expression of iNOS, COX-2, etc., and the production of inflammatory mediators such as NO, PGE2, IL-1, IL-6, and TNF-α [64]. Zhao et al. found that PCP exerts anti-inflammatory and antioxidant effects by activating the ERK/Nrf2/HO-1 signaling pathway in vsmc, decreasing the expression of LOX-1, and eliminating intracellular lipid accumulation [51]. Song et al. found that PCP inhibited RANKL-induced osteoclast formation and bone resorption by down-regulating the phosphorylation levels of STAT3, P38, ERK, and JNK, inhibiting the expression of NFAcT1 and c-Fos, and impacting the expression of TRAcP and CTSK [52]. A study found that PCP exerts a protective effect against fecal-induced peritonitis (FIP) by decreasing inflammatory cytokines and oxidative stress in the plasma, modulating Treg cells in the spleen, and down-regulating Annexin-V in the thymus of septic mice [53]. Another study showed that PCP reduced alcohol-induced liver inflammation by repairing the intestinal barrier and reducing lipopolysaccharide (LPS) leakage, inhibited the TLR4/NF-κB signaling pathway, and improved hepatocyte apoptosis by inhibiting the CYP2E1/ROS/MAPKs signaling pathway [54].

To sum up, *P. cocos* polysaccharides exert their anti-inflammatory efficacy by inhibiting the production of inflammatory mediators and the release of inflammatory factors, activating the ERK/Nrf2/HO-1 signaling pathway, reducing the accumulation of lipids, attenuating oxidative stress, and protecting the intestinal barrier, as well as by inhibiting the TLR4/NF-κB and CYP2E1/ROS/MAPKs signaling pathways.

### 3.3. Anti-Tumor and Immunomodulatory Functions of P. cocos Polysaccharides

*P. cocos* polysaccharides have anti-tumor activity, which may play a role by activating the immune system, inhibiting the proliferation of tumor cells, and promoting apoptosis of tumor cells and other mechanisms [65]. Numerous studies have reported that the anti-tumor activity of *P. cocos* polysaccharides is mainly reflected in two aspects: one is to promote the apoptosis of tumor cells through various mechanisms, thus achieving direct tumor suppression effect; the other is to promote the sensitivity of tumor cells to radiotherapy and chemotherapy, thus achieving an indirect tumor suppression effect [66,67,68]. Polysaccharides can enhance the immune function of the body, activate immune cells such as macrophages and lymphocytes, and promote the production of antibodies, thus improving the body’s resistance to pathogens [69]. Current research on the immunomodulatory function of *P. cocos* mainly focuses on polysaccharide and structurally modified polysaccharide derivatives [70]. *P. cocos* polysaccharides (PCPs) regulate the body’s immunity by protecting the immune organs, preventing or reducing thymus atrophy and spleen enlargement, activating T and B lymphocytes, enhancing NK cell activity, and regulating inflammatory cytokines such as IL-2, TNF-a, and so on [71]. In a study with RAW264.7 cells, Hu et al. found that the homopolysaccharide PCP-1C further exhibited anti-tumor activity in vivo by activating the Notch signaling pathway in macrophages and upregulating the expression of Notch1, and ligands Jagghd1 and Hes1 [55]. Sun et al. found that PCP-W1 enhanced NO, IL-6, IL-1β, TNF- α, and ROS in macrophages by regulating the TLR4/MD2/NF-κB pathway, suggesting that PCP-W1 may have a potential anti-tumor effect [56]. Tian et al. found that the use of PCP led to a significant reduction in tumor volume and an increase in organ immunoreactivity indexes, and it exerted exogenous and internal immunomodulatory effects by activating the TLR4/TRAF6/NF-κB signaling pathway [57].

Interestingly, water-soluble polysaccharides and water-insoluble polysaccharides derived from *P. cocos* have a large difference in immunomodulatory function and anti-tumor activity [72]. Water-soluble polysaccharides are more easily recognized and processed by the immune system to rapidly stimulate an immune response, which is reflected in the enhancement of the body’s immune function through the activation of immune cells [73]. Water-insoluble polysaccharides, on the other hand, exhibit specific anti-tumor activities due to their structural stability and interaction with cell surface receptors, and they can play a role by inhibiting tumor cell proliferation, promoting apoptosis, or affecting the tumor microenvironment [74]. In a study of insoluble polysaccharides, scholars found that PCAPS1 enhanced the mRNA expression levels of TNF-a and nuclear factor jB (NF-jB), while activating RAW264.7 cells by inducing the NF-jB p65 signaling pathway translocation [58]. Lv et al. found that the water-soluble polysaccharide PCP-2 could stimulate the proliferation of RAW264.7 cells, promote the secretion of inflammatory factors such as NO and TNF-α, and increase the levels of IgG, IgA, IgM, and CD3^+^CD4^+^ T cells in the blood [59]. Another study showed that PCP can elicit a strong antibody response in the body by promoting the activation of dendritic cells (DCs) and macrophages in combination with Th1 and Th2 immune responses [60]. In a word, *P. cocos* polysaccharides enhance immune function by activating immune cells and promoting antibody production, and they concurrently exert anti-tumor effects by inhibiting tumor cell proliferation and promoting apoptosis through the activation of signaling pathways such as Notch and TLR4/MD2/NF-κB. Moreover, they can achieve immunomodulation by regulating inflammatory factors and enhancing the function of immune organs, in which water-soluble polysaccharides promote immune responses through rapid activation of the immune system, whereas water-insoluble polysaccharides exert specific anti-tumor activities by affecting the tumor microenvironment and the interaction of cell surface receptors.

In addition to the main pharmacological activities mentioned above, *P. cocos* polysaccharides also have pharmacological effects such as antioxidant, anti-aging, and protection effects on the liver and kidneys [75]. Mechanistic studies have shown that *P. cocos* polysaccharides can elevate superoxide dismutase, which is essential for oxidation and antioxidants in organisms, thus protecting the structure and function of cell membranes from the interference and damage of peroxides and improving the antioxidant capacity of the organism [76,77]. In addition, *P. cocos* polysaccharides inhibited the phosphorylation of mitogen-activated protein kinase p38 antibody (p38MAPK), thereby inhibiting the production of inflammatory factors and thus protecting the kidneys [14]. *P. cocos* polysaccharides protected the liver by down-regulating the expression of TGF01 and PDGF, inhibiting the activation of HSC proliferation, promoting the degradation of the extracellular matrix, and decreasing hepatic fibrous connective tissue deposition [78]. Moreover, *P. cocos* polysaccharides can also be used as prebiotics to widely regulate gut microbiota and improve intestinal barrier function due to their unique activity [79]. Prebiotics are a class of non-digestible macromolecular carbohydrates that produce health benefits for the host by selectively promoting the activity and growth of one or several beneficial bacteria in the gut. A widely used prebiotic are polysaccharides from natural products, which have a variety of positive effects on the health of the host [80,81]. As a powerful prebiotic, *P. cocos* polysaccharides are worth exploring in depth for their multifaceted effects on the maintenance and improvement of intestinal health, helping to improve overall digestive health and the body’s immunity.

### 3.4. P. cocos Polysaccharides Modulate Gut Microbiota to Exert Probiotic Function

With the development of modern biotechnology and pharmacology, polysaccharides have been found to have a wide range of biological functions [82]. The research on the composition, extraction, analysis, and biological activity of *P. cocos* polysaccharides has become a hot spot at home and abroad [83]. Despite extensive reviews on *P. cocos* polysaccharides covering aspects such as extraction processes, structural characterization, anti-tumor activities, anti-inflammatory properties, and immunomodulatory effects, limited attention has been given to summarizing their potential health-promoting effects via modulation of the gut microbiota [84]. Therefore, we will focus on how polysaccharides can ameliorate diseases by modulating the gut microbiota, altering related metabolites, and influencing organismal functions.

**Figure 2:**
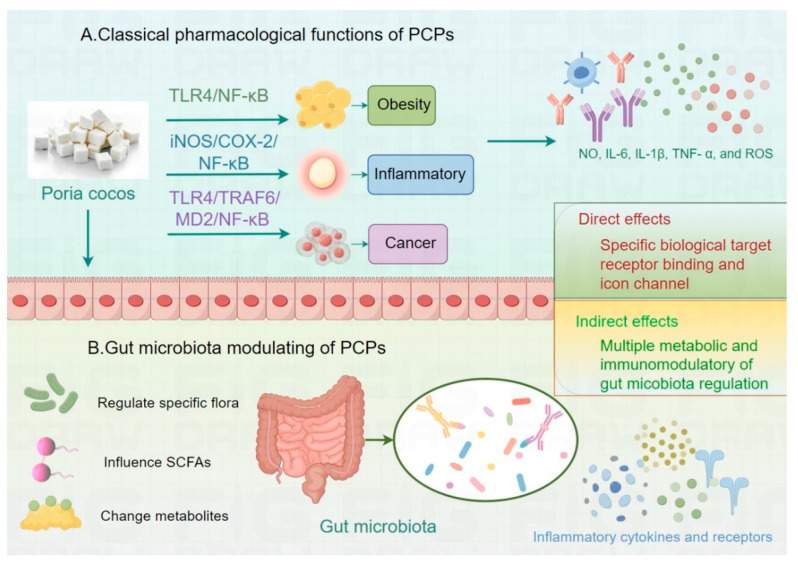
PCPs activate or inhibit the TLR4/NF-κB, CYP2E1/ROS/MAPKs, and TLR4/MD2/NF-κB pathways, releasing cytokines that exert anti-obesity, anti-inflammatory, and anti-tumor effects. In contrast to the above, PCPs affect host metabolic, immune, and inflammatory responses by regulating the gut microbiota, influencing the production of SCFAs, and altering flora-associated metabolites.

Gut microorganisms have a crucial role in human health, and they play a variety of roles in human physiological and pathological processes [85]. The gut microbiota is not only involved in material metabolism and nutrient synthesis but is also closely related to the regulation of the immune system and the development of many diseases [86]. On the one hand, the interaction between gut microbes and the host immune system is crucial for maintaining immune homeostasis [87]. On the other hand, alterations in the composition and function of gut microbes have been associated with a variety of diseases, including diabetes, antibiotic-associated diarrhea, inflammatory bowel disease, and colorectal cancer [88]. Herein, this study of the regulatory effects of *P. cocos* polysaccharides on the gut microbiota is expected to provide new prospects for the effective amelioration of the disease (Figure 2).

#### 3.4.1. Regulate the Composition and Structure of Gut Microbiota

*P. cocos* polysaccharides can regulate the composition and structure of gut microbiota by promoting the growth of beneficial bacteria (*Muribaculaceae* and *Lachnospiraceae*) and inhibiting the reproduction of harmful bacteria (*Escherichia-Shigella*, *Staphylococcus*, and *Acinetobacter*) [89]. The balance of gut microbiota is crucial to human health, and its diversity and stability are key to maintaining intestinal health [90]. Beneficial bacteria such as *Bifidobacteria* and *Lactobacillus* can enhance the intestinal barrier function and inhibit the invasion of pathogenic bacteria [91]. By regulating the gut microbiota, PCPs can help prevent and treat intestinal-related diseases, such as inflammatory bowel disease (IBD) and irritable bowel syndrome (IBS) [92].

#### 3.4.2. Influence on the Production of Short-Chain Fatty Acids (SCFAs)

SCFAs contain acetate, propionate, and butyrate, and are the main metabolites produced by gut microorganisms fermenting cellulose [93]. PCPs can promote the growth of beneficial intestinal bacteria and increase the production of SCFAs [94]. SCFAs are involved in the regulation of energy metabolism and blood glucose control in the host, which helps to prevent and treat metabolic diseases such as obesity and diabetes [95]. Concomitantly, SCFAs exert anti-tumor effects by enhancing immune cell function, directly acting on tumor cells, regulating the tumor microenvironment, and inhibiting HDAC activity [96]. As ligands for histone deacetylases (HDAC), SCFAs can induce HDAC inhibition to stimulate monocytes and neutrophils, leading to NF-κB inactivation and a reduction in inflammatory cytokines [97]. In addition, SCFAs bind to intestinal GPR43 and GPR41 receptors, which further inhibits MAPK, p-p38/p38, and p-JNK/JNK signaling pathways and increases the expression of immune cytokines (e.g., IL-2, IL-4, IL-6, IL-10, TGF-β, and IFN-γ), thus exerting immune activity [98,99].

#### 3.4.3. Regulation of Flora-Associated Metabolites

Gut flora metabolites are key regulators of host health and disease and are diverse and multifunctional, including short-chain fatty acids (SCFAs), bile acids, tryptophan, and trimethylamine-N-oxide (TMAO). *P. cocos* polysaccharides affect tryptophan and bile acid metabolism by regulating the metabolic activity of the gut microbiota, thus positively affecting host health [100]. Tryptophan is one of the essential amino acids, and gut microbes are able to metabolize tryptophan to produce a variety of metabolites such as 5-hydroxytryptophan (serotonin) and kynurenine [101]. These metabolites play an important role in regulating the host’s mood, sleep, and immune response. Bile acids are digestive fluids synthesized by the liver and are involved in the digestion and absorption of fats [102]. Gut microbes metabolize bile acids to produce secondary bile acids, and these metabolites play an important role in regulating the host’s metabolic, immune, and cardiovascular health [103]. On the one hand, *P. cocos* polysaccharides can activate immune effector cells and promote the expression of growth factors and cytokines by regulating the intestinal microbiota and metabolic profile, thus enhancing innate and specific immunity. On the other hand, excessive immune responses can damage tissues and organs, aggravate pathological conditions, and induce autoimmune diseases. By regulating metabolites and immune responses, *P. cocos* polysaccharides can both enhance the body’s immune defense function and avoid organ damage caused by excessive immune responses [104].

Collectively, *P. cocos* polysaccharides act comprehensively on the intestinal microbiota through a variety of mechanisms, including promoting the growth of beneficial bacteria, increasing the production of SCFAs, regulating the intestinal barrier function, influencing the host metabolism, and altering the structure of the bacterial flora, which work together to maintain the intestinal micro-ecological balance and enhance the body’s immune defense ability.

## 4. *P. cocos* Polysaccharides Improve Disease by Regulating Gut Microbiota

Conventional pharmacology usually relies on direct action on specific molecular targets or signaling pathways [105]. The NF-κB pathway plays a key role in immune, inflammatory, and other processes, and its activation mechanism includes both classical and non-classical pathways involving the IκB kinase complex (IKK) and NF-κB-induced kinase (NIK) [64]. In contrast, the paradigm of targeting the modulation of the gut microbiota for disease amelioration focuses on influencing host health by regulating the composition and metabolic activities of gut microbes [106,107]. Typically, the gut microbiota influences the host’s metabolic, immune, and inflammatory responses through the production of metabolites such as SCFAs [18]. Traditional pharmacology focuses on the treatment of specific diseases or symptoms, and the effects of drugs are usually limited to specific organs or systems [108]. In contrast, modulation of the gut microbiota has a broader therapeutic potential, as evidenced by the fact that the gut microbiota is closely related to the function of multiple systems, including metabolism, immunity, and the nervous system [109]. Thus, gut microbiota modulation has a positive impact on a wide range of chronic diseases, and in the next section we will focus on the role of flora modulation in prebiotic function, anti-inflammation, anti-obesity, and immunomodulation (Table 2).

### 4.1. P. cocos Polysaccharides as Dietary Supplements for Health Promotion

Previous studies have demonstrated that prebiotics, as dietary supplements, exert significant effects on modulating the gut microbiota by promoting the proliferation of beneficial bacteria such as Bifidobacterium and Lactobacillus, thereby maintaining gut homeostasis, enhancing digestive function, and strengthening the intestinal barrier [130]. Prebiotics are non-digestible food ingredients that selectively promote the growth and activity of beneficial gut microbiota, thereby improving host health [131]. Polysaccharides exhibited prebiotic effects by modulating the composition of the gut microbiota, reducing the abundance of potentially pathogenic bacteria, and promoting the proliferation of SCFA-producing bacteria. A study shows that polysaccharides from *P. cocos* can function as prebiotics by significantly reshaping the composition of the gut microbiome, thereby contributing to health-promoting effects [110]. Duan et al. found that PCP, with a molecular weight of 11.583 kDa, is a functional food that increases the abundance of *Muribaculaceae* and *Bacteroides*, promotes the production of SCFAs, up-regulates the expression of intestinal Occludin and ZO-1, down-regulates serum endotoxin, DAO, d-lactate, and intestinal MPO levels, and enhances the intestinal barrier by increasing the expression of IL-2, IL-4, IL-6, IL-10, TGF-β, and IFN-γ [111]. In male 21-day-old SD young rats, Wang et al. found that supplementation with PCPs favored the development and metabolic activity of the gut microbiota, and that PCPs increased the relative abundance of beneficial bacteria, decreased the relative abundance of detrimental bacteria, and effectively improved energy metabolism and nucleic acid metabolism [112]. A comparative study of *P. cocos* water-soluble polysaccharides (PCX) and water-insoluble polysaccharides (PCY) found that both were effective in modulating the gut microbiota of KM mice and altering short peptide metabolism and inflammatory factors, especially PCY, which has great prebiotic potential [113].

### 4.2. P. cocos Polysaccharides Exert Anti-Inflammatory Effects to Improve Disease

Modern pharmacological studies have demonstrated that *P. cocos* polysaccharides exert their anti-inflammatory effect by affecting the secretion of inflammation-related factors, iNOS and COX-2, and inhibiting the activation of the inflammatory signaling pathway NF-κB [132]. It has been shown that in DSS-induced ulcerative colitis, *P. cocos* oligosaccharides protect the intestinal barrier from damage by regulating the relative abundance of *Muribaculum*, *Desulfovibrio*, *Oscillibacter*, *Escherichia*-*Shigella*, and *Turicibacter*, inhibiting the expression of TNF-α, IL-1β, and lL-6 cytokines, and promoting the expression of tight junction proteins to protect the intestinal barrier from damage [114]. *P. cocos* aqueous polysaccharides improved IBD by significantly decreasing the number of *Pseudomonas* and increasing the abundance of *Oscillospira*, *Prevotella*, *Ruminococcus*, and unidentified-*Lachnospiraceae*, inhibiting the NF-κB pathway, decreasing inflammatory mediators, and restoring the expression of colonic tight junction proteins ZO-1 and Claudin-1 expression [115]. The gut microbiota affects the immune system, metabolic processes, and intestinal barrier function by influencing the immune system, metabolic processes, and intestinal barrier function in the treatment of chronic nonbacterial prostatitis (CNP). Therefore, natural product polysaccharides modulating the gut microbiota may provide a new strategy for the treatment of CNP [133,134]. *P. cocos* polysaccharides (PPs) reduce CNP symptoms by enriching *Parabacteroides* and *Fusicatenibacter*, increasing beneficial metabolites in the gut microbiome, and upregulating the expression of Alox15 and Pla2g2f in the colonic epithelium to inhibit prostate inflammation [116]. In a study of CNP, Liu et al. found that polysaccharide PPs over-reduced the levels of pro-inflammatory cytokines (TNF-α and IL-1β) and c-reactive protein (CRP), while PPs restored the relative abundance of five genera, reconfigured the DNA methylome of intestinal epithelial cells, and played a key role in alleviating CNP [117]. Furthermore, in their another comparative study of PPs with finasteride in the treatment of CNP, both PPs and finasteride inhibited the production of pro-inflammatory cytokines and androgens, and interfered with CNP by restoring CNP-induced changes in the bacterial flora, including *Ruminococcaceae_NK4A214_group, bacterium_f_Ruminococcaceae, Ruminiclostridium_9*, *Phascolarctobacterium, Coriobacteriaceae UCG-002,* and *Oribacterium* [118].

### 4.3. P. cocos Polysaccharides Effectively Alleviate Metabolic Syndrome

The metabolic syndrome, typified by obesity, hyperglycaemia and hepatic steatosis, is prevalent worldwide [135]. Metabolites produced by the gut microbiota, such as trimethylamine and secondary bile acids, are involved in the body’s glycolipid metabolism and inflammatory response, influencing the development of the metabolic syndrome [45]. Studies have shown that modulating the gut microbiota with prebiotics, probiotics, or fecal mushroom transplantation (FMT) can help improve the symptoms of metabolic syndrome [136]. Sun et al. found that WIP with the repeating unit (1–3)-β-D-glucan modulated the amount of *Lachnospiracea* and *Clostridium*, increased the level of butyrate in the intestine, improved the integrity of the intestinal mucosa, and activated the PPAR-γ pathway [119]. A comprehensive analysis of the Jiang et al. study found that PCP altered the amount of *Eisenbergiella*, *Dorea*, *Proteiniphilum*, and *Lachnospira* in the gut, and inhibited inflammatory signaling pathways in adipose tissue induced by a high-fat diet [120]. In addition, Zhu et al. showed that PCO could be used as a new prebiotic, and that PCO inhibited the expression of fatty acid synthesis regulator mRNA and pro-inflammatory cytokines, restored the imbalance of the intestinal microbiota in the HFD mice, and reversed a variety of intestinal metabolites [121]. A study demonstrated that PCP significantly alleviated histological liver injury and impaired liver function in C57BL/6 mice, suggesting that PCP modulates the gut microbiota and downregulates the NF-κB/CCL3/CCR1 axis to ameliorate non-alcoholic steatohepatitis [122]. Another study found that WIP from *P. cocos* treated intestinal fungal-induced PGE2-induced alcoholic hepatic steatosis by activating PPAR-γ signaling, decreasing colonic inflammation, increasing the relative abundance of *Ruminoclostridum* and *unidentified_clostridials*, and inhibiting the overgrowth of fungal and *Proteobacteria* in the intestine [123].

### 4.4. P. cocos Polysaccharides Have Superior Immunomodulatory Functions

*P. cocos* polysaccharides have a variety of biological activities, especially in immunomodulation, showing great potential to improve a variety of diseases [20]. *P. cocos* polysaccharides can activate macrophages, increase the lymphocyte conversion rate and natural killer cell activity, promote T lymphocyte proliferation, regulate cytokine secretion, and enhance the body’s cellular and humoral immune functions [137]. Zhang et al. found that PCWP ameliorated sleep deprivation-induced anxiety by modulating intestinal ecological dysregulation, inhibiting the TNF-α/NF-κB signaling pathway, and alleviating metabolic disturbances [124]. Ye et al. found that PCP exerts an immunomodulatory function and is involved in regulating the composition, function, and metabolism of the gut and lung microbiota to reverse CSA-induced immunosuppressive lung injury [125]. Yin et al. found that PCP attenuated the adverse effects of 5-FU and improved therapeutic efficacy in *Apc^Min^*^/+^ mice, and they verified that PCP had a prebiotic effect on enhancing 5-FU by transplanting *Lactobacillus johnsonii* and *Bifdobacterium animalis*. In their study of cisplatin damage to intestinal immunity [126]. Zou et al. found that WP attenuated intestinal injury by modulating the intestinal microbiota and metabolic profile and reversing the elevation of inflammatory mediators [127].

In addition, antibiotic-associated diarrhea is a common and serious problem resulting from the clinical misuse of antibiotics, and its typical diarrhea features are usually accompanied by disturbances in the gut microbiota [138]. Reversing the imbalance in the gut microbiota is one of the most effective treatments for improving AAD, and the intake of polysaccharide prebiotics restores the balance of the flora by increasing the beneficial bacteria and decreasing the pathogenic bacteria [139]. Thus, PCPs ameliorate AAD in mice by modulating the homeostasis of the intestinal microbiota and the intestinal mucosal barrier, showing their potential prebiotic effects and therapeutic efficacy in host immune response and metabolic function [140]. Xu et al. showed that PCP could improve AAD in mice by modulating the homeostasis of the gut microbiota and the intestinal mucosal barrier, by increasing the expression level of the colonic tight junction protein ZO-1, and by regulating the expression of the FOXP3 and GPR41 mRNAs [128]. Interestingly, a study by Lai et al. demonstrated that the PCY originating from *P. cocos* could also alleviate AAD symptoms by increasing the relative abundance of *Muribaculaceae* and *Lachnospiraceae*, decreasing the relative abundance of *Escherichia*-*Shigella*, *Staphylococcus*, and *Acinetobacter*, promoting the production of SCFAs and decreasing inflammatory response [129].

## 5. Conclusions and Future Perspective

In this study, we provide information on the bioactivities, extraction methods, structural characterization, and chemical modifications of *P. cocos* and its major ingredients, explore the pharmacological effects of *P. cocos* polysaccharides including anti-obesity, anti-inflammatory, anti-tumor, and immunomodulatory functions, and systematically review the scientific evidence for the modulatory effects of *P. cocos* polysaccharides on the gut microbiota and their health benefits. The conformational relationships of polysaccharides are characterized by their molecular weights, monosaccharide compositions, glycosidic bond types, and high-level structures, which together affect the biological activities, and an in-depth understanding of these conformational relationships is of great significance for the development of natural polysaccharides [141,142,143]. It is worth noting that *P. cocos* polysaccharides can be categorized into water-soluble polysaccharides and alkali-soluble polysaccharides according to their differences in structure and biological activity. Water-soluble *P. cocos* polysaccharides are more easily absorbed by the body due to their good solubility, and they have excellent biological activities such as anti-tumor, anti-inflammatory, and immunomodulatory effects. The water-insoluble polysaccharides, mainly β-(1 → 3)-D-glucan, are more abundant in *P. cocos*, but have poor water solubility and lower activity than water-soluble polysaccharides [144]. Thus, researchers usually adopt methods, such as degrading polysaccharides into oligosaccharides, or carboxymethylating and sulfating them, to improve water solubility and biological activity [145]. Polysaccharides have been demonstrated to positively impact health on multiple levels by regulating the gut microbiota [146]. As research continues, the relationship between the targets of *P. cocos* polysaccharides acting on the gut microbiota and diseases will be more clearly elucidated [147]. The role of *P. cocos* polysaccharides in improving diseases by targeting the gut microbiota is mainly concentrated on anti-inflammatory, anti-obesity, immune modulation, and prebiotic effects due to the specificity of microbial regulation [148,149]. In this review, we learn that the bioactivities of *P. cocos* polysaccharides targeting the gut microbiota are both related to and different from traditional pharmacological functions. Moreover, the modulatory effects of *P. cocos* polysaccharides on the gut microbiota involve improvement in intestinal barrier function and a reduction in intestinal inflammation via SCFAs, which provides an indirect, yet more integrated and stable, therapeutic approach to treating related diseases. Herein, the main difference between the above two strategies lies in the directness and indirectness of the pathways, as well as the difference in therapeutic focus, with the former indirectly affecting the metabolic and immune status of the host through the modulation of the intestinal microbiota, whereas the latter is usually directly directed to a specific biological target.

In conclusion, this paper reviews the health benefits of *P. cocos* polysaccharides based on the modulation of the gut microbiota, and it emphasizes the relationship between flora modulation and biological functions. Future studies need to combine multidisciplinary knowledge from microbiology, nutrition, immunology, and systems biology to comprehensively understand the regulatory mechanisms of *P. cocos* polysaccharides on the gut microbiota and their integrated effects on human health. This review contributes to the further development of *P. cocos* as a functional product and also provides ideas and insights for developing new therapeutic strategies.

## Figures and Tables

**Table 1:** Extensive pharmacological functions of PCPs and their mechanisms.

Name	Structure	Dosage	Model	Pharmacological Function	Molecular Mechanisms and Targets	Symptoms of Improvement	Reference
PCP	PCPs (95% pure) were purchased	100 mg/kg, 200 mg/kg, and 400 mg/kg	Male 8-week C57/BL6 mice and *ApoE*^−/−^ mice	Reduce high-fat diet-induced arteriosclerosis	PCP inhibited the activation of the TLR4/NF-κB pathway in the aorta, serum inflammatory mediators and lipids, and blocked the expression of matrix metalloproteinase 2 and intercellular adhesion molecule 1 proteins.	PCP intervenes in AS by reducing inflammatory factors and blood lipid levels.	[48]
PCP	*N*	225 mg/kg	Male 8-week C57/BL6 mice	Improve lipid metabolism to alleviate NAFLD	PCP affects lipid synthesis, uptake, and oxidation by regulating the expression of genes related to bile acid synthesis (*Cyp7b1* and *Cyp27a1*) and fatty acid metabolism (*Srebp1c*, *Fas*, *Scd1*, *Fatp2*, *Pparα*).	PCP reduced serum and hepatic lipid levels, and improved lipid metabolism by regulating bile acids and fatty acid metabolism.	[49]
PCO	(1 → 2)-β-D-Glcp, (1 → 2)-α-D-Glcp, and (1 → 4)-α-D-Glcp.	200 mg/kg	Male 6-week C57/BL6 mice	Improve HFD-induced dyslipidemia	PCO reversed the expression of bile acid synthesis *genes* (*CYP7A1*, *CYP8B1*, *CYP27A1*, *ABCG1*) to promote lipid metabolism and reduced the mRNA levels of inflammatory cytokines to attenuate inflammatory responses in mouse liver.	PCO inhibits lipid metabolism disorders and reduces lipid accumulation and inflammatory responses in blood and liver tissues.	[50]
PCP	*N*	50, 100, and 200 μg/mL	Vascular smooth muscle cells (VSMCs)	Attenuate ox-LDL-induced inflammation and oxidative stress	PCP activate the ERK/Nrf2/HO-1 signaling pathway in vsmc, decreasing LOX-1 expression, and eliminating intracellular lipid accumulation.	PCP exerts anti-inflammatory effects by inhibiting pro-inflammatory mediators and cytokines and ameliorates oxidative stress.	[51]
PCP	Purity > 98%	0.5–8 µM	RAW264.7 cells	Attenuate RANKL induced osteoclastogenesis	PCP down-regulated the phosphorylation levels of STAT3, P38, ERK, and JNK, and inhibited the expression of NFAcT1 and c-Fos, and TRAcP and CTSK.	PCP inhibits RANKL-induced osteoclast formation and bone resorption.	[52]
PCP	Purity > 90%	200 mg/kg, and 400 mg/kg	Male 6–8-week C57/BL6 mice	Improve fecal-induced peritonitis (FIP)	PCP regulates Treg cells in the spleen and downregulates Annexin-V in the thymus of fp-induced sepsis mice.	PCP reduced inflammatory cytokines and oxidative stress in the plasma and spleen and increased resistance to FIP.	[53]
PCP-1C	*N*	25, 50, and 100 mg/kg	Male C57/BL6 mice	Improve alcohol-induced liver injury	PCP-1C reduced hepatic inflammation by inhibiting the TLR4/NF-κB signaling pathway and ameliorated hepatocyte apoptosis by inhibiting CYP2E1/ROS/MAPKs signaling pathway.	PCP-1C reduces serum biochemical markers, attenuates hepatic steatosis, repairs the intestinal barrier, and reduces lipopolysaccharide (LPS) leakage.	[54]
PCP-1C	Sugar content: 96.97%	50, 100, and 200 µg/mL	RAW264. 7 cells	Regulate the immune response and anti-tumor	PCP-1C activated the Notch signaling pathway in macrophages and up-regulated the expression of Notch1, ligands Jagghd1 and Hes1.	PCP-1C improves M1 macrophage polarization.	[55]
PCP-W1	molecular weight: 18.38 kDa	25, 50, 100, 200 and 400 μg/mL	RAW264. 7 cells	Potential anti-tumor effects	PCP-W1 regulated the TLR4/MD2/NF-κB pathway and activated the release of NO, IL-6, IL-β, TNF-α, CD86, and ROS.	PCP-W1 induced macrophage polarization to m1-type in RAW 264.7 mice, and	[56]
PCP	>90% pure	200 μg/mL for cell and 200 mg/kg for mice	RAW 264.7 cells, female C57BL/10ScNJ, and control C57BL/10J mice	Immunomodulatory effects	PCP elevated the levels of nitric oxide, IL-2, IL-6, IL-17 A, TNF, and IFN-γ and significantly increased the expression of TLR4, MyD88, TRAF-6, p-NF-κB, and p-c-JUN.	PCP led to a significant decrease in tumor volume and an increase in all organ immunoreactivity indices.	[57]
PCAPS1	β-1,3-glucan with 11.5 kDa	500 μg/mL	RAW264. 7 cells	Immunoregulatory capacity	PCAPS1 enhanced the mRNA expression levels of TNF-a and NF-jB, activating RAW264.7 cells by inducing translocation of the NF-jB p65 signaling pathway.	PCAPS1 increased the secretion of TNF-a.	[58]
PCP-2	1,3-β-D-Glc and 1,6-β-D-Glc with 2.35 kDa	2, 10, 50, 200 μg/mL	RAW264. 7 cells	Immunoenhancing effects	PCP-2 promoted the secretion of NO and TNF-α, and increased the levels of IgG, IgA, IgM, and CD3^+^CD4^+^ T cells in the blood.	PCP-2 promoted the development of thymus and spleen immune organs, and improved gut barrier dysfunctions.	[59]
PCP	20.112 kDa	50, 200, 800 μg/mL	BALB/c mice	Inducing immune responses	PCP promotes the activation of dendritic cells and macrophages and binds to Th1 and Th2 immune responses.	PCP markedly induced the cytotoxic T lymphocyte response, and enhanced humoral and cellular immune responses.	[60]

**Table 2:** The role of PCPs in regulating gut microbiota and its mechanism.

Name	Dosage	Model	Activity	Gut Microbiota Regulation	Metabolites Regulation	Gene and Protein Expression	Reference
PC	750 mg/kg	Male 6-week C57BL/6 mice	Prebiotic functions	*Barnesiella*, *Pseudobutyrivibrio*, and *Dehalobacterium* were decreased.	PC promoted the production of Bacteroides xylanolyticus.	SCFA affects signaling pathways in host intestinal epithelial cells.	[110]
PCP	75 mg/kg, 150 mg/kg, and 300 mg/kg	Male 8-week C57BL/6 mice	Functional food to regulate intestinal mucosal function	PCP treatment increased the abundance of *Muribaculaceae* and *Bacteroides.*	PCP showed significantly higher levels of acetic acid, propionic acid, isobutyric acid, and isovaleric acid.	PCP up-regulates intestinal Occludin and ZO-1 proteins, MUC2, β-defensin and SIgA, and enhances immunity by up-regulating the expression of IL-2, IL-4, IL-6, IL-10, TGF-β and IFN-γ.	[111]
PCPs	12 g/kg	Male 21-day-old Sprague Dawley rats	Functional food to help promote maturation and stability in young rats	The relative abundances of *Bifidobacterium, Lactobacillus*, *Allobaculum*, and *Oligella* increased, whereas *Enterococcus* declined.	Improved amino acid, energy, SCFAs and nucleotide metabolism. 35 urinary and 24 feces metabolites was changed.	PCPs could promote the functional maturation of gut microbiota and increase basic metabolism.	[112]
PCX	300 mg/kg	Male 4–6-week Kunming mice	Functional prebiotic	PCX increased *Lachnospiraceae* and decreased *Muribaculaceae.*	The content of short-chain peptides was changed.	PCX significantly elevated IL-10 levels.	[113]
PCY	300 mg/kg	Male 4–6-week Kunming mice	Functional prebiotic	The number of *Lactobacillus* was significantly increased.	The content of short-chain peptides was changed.	PCY significantly decreased IFN-γ levels.	[113]
PCE	20.46 mg/kg, 40.92 mg/kg, 81.84 mg/kg	Male 6–8-week Balb/c mice	Attenuated DSS-induced ulcerative colitis	PCE significantly decreased *Pseudomonas*, and increased *Oscillospira*, *Prevotella*, *Ruminococcus*, and unidentified-*Lachnospiraceae.*	*N*	PCE inhibited the NF-κB pathway, decreased the inflammatory mediators TNF-α, IL-6, and IL-1β, and restored the expression of the colonic tight junction proteins ZO-1 and Claudin-1.	[114]
PCOs	200 mg/kg	Male 6-week BALB/c mice	Attenuated DSS-induced ulcerative colitis	Regulated the relative abundance of *Muribaculum*, *Desulfovibrio*, *Oscillibacter*, *Escherichia–Shigella*, and *Turicibacter.*	Secondary bile acid biosynthesis, the sulfur relay system, and glutathione metabolism were remarkably reversed.	PCOs protect the intestinal barrier from damage by inhibiting TNF-α, IL-1β, and lL-6 cytokines and promoting the expression of tight junction proteins.	[115]
PPs	250 mg/kg	Male Sprague Dawley (SD) 7–8-week rats	Alleviated Chronic nonbacterial prostatitis	*Parabacteroides*, *Fusicatenibacter*, and *Parasutterella* were significantly enriched.	PPs increased beneficial metabolites 7-ketodeoxycholic acid and haloperidol glucuronide.	The expression of Alox15 and Pla2g2f increased, and Cyp1a1 and Hsd17b7 reduced in colon epithelium.	[116]
PPs	100 mg/kg, 250 mg/kg, 500 mg/kg	Male Sprague Dawley (SD) 7–8-week rats	Alleviated Chronic non-bacterial prostatitis	*Lachnospiraceae*-NK4A136 group, *Lactobacillus*, and uncultured *bacterium_f_ Erysipelotrichaceae* were decreased, while *Romboutsia* and uncultured *bacterium_f_Desulfovibrionaceae* were increased.	Altered the levels of malondialdehyde (MDA) and superoxide di-uronidase (SOD).	PPs exert anti-CNP effects by decreasing the levels of NF-α and IL-1β and c-reactive protein (CRP) and modulating the production of testosterone (T), dihydrotestosterone (DTH), and estradiol (E2), nitric oxide synthase (iNOS).	[117]
PPs	250 mg/kg	Male Sprague Dawley (SD) 7–8-week rats	Alleviated chronic non-bacterial prostatitis	Restored CNP-induced changes in the bacterial flora, including *Ruminococcaceae NK4A214 group*, *uncultured bacterium_f_Ruminococcaceae*, *Ruminiclostridium_9*, *Phascolarctobacterium*, *Coriobacteriaceae UCG-002* and *Oribacterium*.	*N*	Inhibited the production of pro-inflammatory cytokines (TNF-α, IL-2 and IL-8) and androgens (dihy_x005f drotestosterone and testosterone).	[118]
WIP	1 g/kg and 0.5 g/kg	Ob/ob 8-week mice	Improved hyperglycemia, hyperlipidemia and hepatic steatosis	WIP promoted an increase in *Lachnospiracea* and *Clostridium.*	WIP treatment ele_x005f vated the level of butyrate in gut.	Improved gut mucosal integrity and activated the intestinal PPAR-γ pathway.	[119]
PCP	100 mg/kg	Male 6-week C57BL/6 mice	Anti-obesity effect	Altered *Eisenbergiella*, *Dorea*, *Proteiniphilum* and *Lachnospira* in response to PCP treatment.	PCP treatment improved vitamin B6 metabolism and ubiquitin system function.	PCP improves fatty acid metabolism and mitigates intestinal barrier disruption induced by HFD in vivo.	[120]
PCO	200 mg/kg	Male 6-week C57BL/6 mice	Improved glycolipid metabolism disturbance	PCO decreased *Ruminococx_0002_caceae* and *Anaeroplasmataceae*, and increased *Lactobacillaceae* and *Rikenellaceae.*	Reverse bile acids (BAs), short-chain fatty acids (SCFAs), and tryptophan metabolites.	PCO inhibited the expression of fatty acid synthesis regulator mRNA and pro-inflammatory cytokines.	[121]
PCP	150 mg/kg and 300 mg/kg	Male 9-week C57BL/6 mice	Improved non-alcoholic steatohepatitis	PCP significantly increasing the relative abundance of *Faecalibaculum.*	Decreased the level of endotoxin load derived from gut bacteria.	PCP regulates the expression of the chemokine, Toll-like receptor, and the NF_x0002_kappa B signaling pathway.	[122]
WIP	1.0 g/kg	Male 8-week C57BL/6 mice	Improved gut fungi-induced PGE2 to alcoholic hepatic steatosis	WIP significantly enhanced the ratio of Firmictues to Proteobacteria, and increased *Lachnospiraceae*, *Ruminoclostridum*, and unidentified_*clostridials*	*N*	WIP ameliorates hepatic inflammatory injury and fat accumulation by activating PPAR-γ signaling and reducing colonic inflammation.	[123]
PCWP	100 mg/kg	Male 8-week Wistar rats	Modulated anxiety-like behavior induced by sleep deprivation	Adjusted the abundance of *Rikenellaceae_RC9_ gut_group*, *Ruminococcus*, *Prevotellaceae_UCG-001*, *Prevotellaceae*, and *Fusicatenibacter.*	PCWP intervention moderated sphingolipid, phenyl_x0002_alanine, and taurine and hypotaurine metabolism.	PCWP regulated gastrointestinal peptide levels, reduced inflammatory factors, and inhibited the TNF-α/NF-κB signaling pathway.	[124]
PCP	100 mg/kg	Half male and female BLAB/c mice	Ameliorated CsA-induced immunosuppressive lung injury	PCP intervention significantly reduced the abundance of *Chryseobacterium*, *Lawsonella*, *Paracoccus*, and *Sediminibacterium*, and increased *Alloprevotella.*	The model serum metabolite Americine decreased the expression of PC(O-18:1(4Z)/0:0).	PCP restored organ indices and lung tissue morphology and structure.	[125]
PCP	750 mg/kg	6–8 weeks *Apc^Min^*^/+^ mice	Attenuated the adverse effects of 5-FU	PCP stimulated the growth of *Bacteroides acidifaciens*, *Bacteroides intestinihominis*, *Butyricicoccus pullicaecorum*, and the genera *Lactobacillus*, *Bifdobacterium*, and *Eubacterium.*	*N*	Reduced the expressions of pro-inflammatory cytokines and enhanced the tight junction proteins and associated adhesion molecules.	[126]
WP	7.6 mg/kg	Male 6–8 weeks C57BL/6 mice	Acted against cisplatin-induced intestinal damage	WP decreased *Proteobacteria*, *Cyanobacteria*, *Ruminococcaceae,* and *Helicobacteraceae*, while promoting *Erysipelotrichaceae* and *Prevotellaceae.*	altered metabolic profiles including xanthine, L-tyrosine, uridine, hypoxanthine, butyrylcarnitine, ribose, plamitic acid, thiamine monophosphate, and indolelactic acid.	WP alleviated weight loss and reversed the elevation of IL-2 and IL-6 in serum.	[127]
PCP	250 mg/kg	Half males and half female 5-week C57BL/6N mice	Ameliorated antibiotic-associated diarrhea	Regulation of seven characteristic species including *Salmonella*, *Parabacteroides*, *Clostridium*, *Ruminoc-occus Lactobacillus*, and *Mucispirillum.*	*N*	PCP increased the expression level of the colonic tight junction protein-occlusion band 1 (ZO-1) and regulated the mRNA expression of FOXP3 and GPR41.	[128]
PCY	300 mg/kg	Male 6-week C57BL/6 mice	Ameliorated antibiotic-associated diarrhea	PCY increased norank_f_*Muribaculaceae* and unclassified_f_*Lachnospiraceae*, and decreased *Escherichia-Shigella*, *Staphylococcus,* and *Acinetobacter*.	The level of acetic and butyric acid in the PCY group was significantly higher.	PCY restored intestinal barrier function and decreased the concentrations of TNF-α, IL-6, and IL-1β.	[129]

## Data Availability

No new data were created or analyzed in this study. Data sharing is not applicable.

## References

[1] Yang Z., Su C., Xu Z., Liu Y., Chen J., Wu X. (2024). Mechanistic and Functional Studies on the Microbial Induction of *Wolfiporia cocos* Liquid Fermentation Products. Foods.

[2] Liang D., Yong T., Diao X., Chen S., Chen D., Xiao C., Zuo D., Xie Y., Zhou X., Hu H. (2021). Hypouricaemic and nephroprotective effects of *Poria cocos* in hyperuricemic mice by up-regulating ATP-binding cassette super-family G member 2. Pharm. Biol..

[3] Yuan J., Hu Y., Yang D., Zhou A., Luo S., Xu N., Dong J., He Q., Zhang C., Zhang X. (2024). The Effects of *Crataegus pinnatifida* and *Wolfiporia extensa* Combination on Diet-Induced Obesity and Gut Microbiota. Foods.

[4] Kim J.H., Sim H.A., Jung D.Y., Lim E.Y., Kim Y.T., Kim B.J., Jung M.H. (2019). *Poria cocus* Wolf Extract Ameliorates Hepatic Steatosis through Regulation of Lipid Metabolism, Inhibition of ER Stress, and Activation of Autophagy via AMPK Activation. Int. J. Mol. Sci..

[5] Tong Q., Chang Y., Shang G., Yin J., Zhou X., Wang S., Yan X., Zhang F., Wang S., Yao W. (2024). Integrated chemical characterization, metabolite profiling, and pharmacokinetics analysis of Zhijun Tangshen Decoction by UPLC-Q/TOF-MS. Front. Pharmacol..

[6] Zong Y., Meng J., Mao T., Han Q., Zhang P., Shi L. (2023). Repairing the intestinal mucosal barrier of traditional Chinese medicine for ulcerative colitis: A review. Front. Pharmacol..

[7] Zhu L., Wang X., Li S., Qi E.R., Meng J., Ching Lam K.Y., Dong X., Xu J., Chen H., Zhao Z. (2020). Qualitative and quantitative characterization of carbohydrate profiles in three different parts of *Poria cocos*. J. Pharm. Biomed. Anal..

[8] Park M., Yi J.-M., Kim N.S., Lee S.-Y., Lee H. (2024). Effect of *Poria cocos* Terpenes: Verifying Modes of Action Using Molecular Docking, Drug-Induced Transcriptomes, and Diffusion Network Analyses. Int. J. Mol. Sci..

[9] Cheng Y., Xie Y., Ge J.C., Wang L., Peng D.Y., Yu N.J., Zhang Y., Jiang Y.H., Luo J.P., Chen W.D. (2021). Structural characterization and hepatoprotective activity of a galactoglucan from *Poria cocos*. Carbohydr. Polym..

[10] Fang C.L., Paul C.R., Day C.H., Chang R.L., Kuo C.H., Ho T.J., Hsieh D.J., Viswanadha V.P., Kuo W.W., Huang C.Y. (2021). *Poria cocos* (Fuling) targets TGFbeta/Smad7 associated collagen accumulation and enhances Nrf2-antioxidant mechanism to exert anti-skin aging effects in human dermal fibroblasts. Environ. Toxicol..

[11] Yasuda S., Okahashi N., Tsugawa H., Ogata Y., Ikeda K., Suda W., Arai H., Hattori M., Arita M. (2020). Elucidation of Gut Microbiota-Associated Lipids Using LC-MS/MS and 16S rRNA Sequence Analyses. iScience.

[12] Lai Y., Fang Q., Guo X., Lei H., Zhou Q., Wu N., Song C. (2022). Effect of polysaccharides from Dictyophora indusiata on regulating gut microbiota and short-chain fatty acids in mice. J. Food Meas. Charact..

[13] Lai Y., Deng H., Chen M., Fan C., Chen Y., Wang F., Zhou Q., Song C. (2023). In vitro fermentation properties of grape seed polysaccharides and the effect on regulating gut microbiota in mice. J. Food Meas. Charact..

[14] Liu C., Du P., Guo Y., Xie Y., Yu H., Yao W., Cheng Y., Qian H. (2021). Extraction, characterization of aloe polysaccharides and the in-depth analysis of its prebiotic effects on mice gut microbiota. Carbohydr. Polym..

[15] Fang Q., Lai Y., Zhang D., Lei H., Wang F., Guo X., Song C. (2023). Gut microbiota regulation and prebiotic properties of polysaccharides from *Oudemansiella raphanipes* mushroom. World J. Microbiol. Biotechnol..

[16] Yin J., Ren W., Wei B., Huang H., Li M., Wu X., Wang A., Xiao Z., Shen J., Zhao Y. (2020). Characterization of chemical composition and prebiotic effect of a dietary medicinal plant *Penthorum chinense* Pursh. Food Chem..

[17] Cheung M.K., Yue G.G.L., Chiu P.W.Y., Lau C.B.S. (2020). A Review of the Effects of Natural Compounds, Medicinal Plants, and Mushrooms on the Gut Microbiota in Colitis and Cancer. Front. Pharmacol..

[18] Feng Y.L., Cao G., Chen D.Q., Vaziri N.D., Chen L., Zhang J., Wang M., Guo Y., Zhao Y.Y. (2019). Microbiome-metabolomics reveals gut microbiota associated with glycine-conjugated metabolites and polyamine metabolism in chronic kidney disease. Cell Mol. Life Sci..

[19] Ng C.Y.J., Lai N.P.Y., Ng W.M., Siah K.T.H., Gan R.-Y., Zhong L.L.D. (2024). Chemical structures, extraction and analysis technologies, and bioactivities of edible fungal polysaccharides from *Poria cocos*: An updated review. Int. J. Biol. Macromol..

[20] Jiang Y., Fan L. (2021). The effect of *Poria cocos* ethanol extract on the intestinal barrier function and intestinal microbiota in mice with breast cancer. J. Ethnopharmacol..

[21] Li L., Zuo Z.-T., Wang Y.-Z. (2022). The Traditional Usages, Chemical Components and Pharmacological Activities of *Wolfiporia cocos*: A Review. Am. J. Chin. Med..

[22] Yang J., Dong X., Li B., Chen T., Yu B., Wang X., Dou X., Peng B., Hu Q. (2023). *Poria cocos* polysaccharide—Functionalized graphene oxide nanosheet induces efficient cancer immunotherapy in mice. Front. Bioeng. Biotechnol..

[23] Ríos J.-L., Andújar I., Recio M.-C., Giner R.-M. (2012). Lanostanoids from Fungi: A Group of Potential Anticancer Compounds. J. Nat. Prod..

[24] Wang D., Huang C., Zhao Y., Wang L., Yang Y., Wang A., Zhang Y., Hu G., Jia J. (2020). Comparative Studies on Polysaccharides, Triterpenoids, and Essential Oil from Fermented Mycelia and Cultivated Sclerotium of a Medicinal and Edible Mushroom, *Poria Cocos*. Molecules.

[25] Chao C.L., Huang H.W., Su M.H., Lin H.C., Wu W.M. (2021). The Lanostane Triterpenoids in *Poria cocos* Play Beneficial Roles in Immunoregulatory Activity. Life.

[26] Wang H., Luo Y., Chu Z., Ni T., Ou S., Dai X., Zhang X., Liu Y. (2022). Poria Acid, Triterpenoids Extracted from *Poria cocos*, Inhibits the Invasion and Metastasis of Gastric Cancer Cells. Molecules.

[27] Meng Y., Hu C., Cheng J., Qiu W., Wang Q., Chen X., Chang C., Hu J., Qiu Z., Zheng G. (2023). The extraction, structure characterization and hydrogel construction of a water-insoluble β-glucan from *Poria cocos*. Carbohydr. Res..

[28] Xu T., Zhang H., Wang S., Xiang Z., Kong H., Xue Q., He M., Yu X., Li Y., Sun D. (2022). A review on the advances in the extraction methods and structure elucidation of *Poria cocos* polysaccharide and its pharmacological activities and drug carrier applications. Int. J. Biol. Macromol..

[29] Zhang W., Cheng S., Zhai X., Sun J., Hu X., Pei H., Chen G. (2020). Green and Efficient Extraction of Polysaccharides From *Poria cocos* F. A. Wolf by Deep Eutectic Solvent. Nat. Prod. Commun..

[30] Guo Y., Li Y., Li Z., Yan W., Chen P., Yao S. (2021). Extraction assisted by far infrared radiation and hot air circulation with deep eutectic solvent for bioactive polysaccharides from *Poria cocos* (Schw.) *wolf*. Green. Chem..

[31] Liang J., Zhao M., Xie S., Peng D., An M., Chen Y., Li P., Du B. (2022). Effect of steam explosion pretreatment on polysaccharide isolated from *Poria cocos*: Structure and immunostimulatory activity. J. Food Biochem..

[32] Yan B., Chen T., Tao Y., Zhang N., Zhao J., Zhang H., Chen W., Fan D. (2024). Fabrication, Functional Properties, and Potential Applications of Mixed Gellan–Polysaccharide Systems: A Review. Annu. Rev. Food Sci. Technol..

[33] Chen C.-Y., Zhang R., Zhang L.-J., Hu Z.-Y., Wang S.-P., Mei X., Mi W., Zhang J.-Y. (2023). Biotransformation and bioaccessibility of active ingredients from Radix Astragali by *Poria cocos* during solid-state fermentation and in vitro digestion and antioxidant activity evaluation. Sci. Rep..

[34] Huang Q., Bao Q., Wu C., Hu M., Chen Y., Wang L., Chen W. (2022). Carbon dots derived from *Poria cocos* polysaccharide as an effective “on-off” fluorescence sensor for chromium (VI) detection. J. Pharm. Anal..

[35] Nie A., Chao Y., Zhang X., Jia W., Zhou Z., Zhu C. (2020). Phytochemistry and Pharmacological Activities of *Wolfiporia cocos* (F. A. Wolf) Ryvarden & Gilb. Front. Pharmacol..

[36] Sun Y. (2014). Biological activities and potential health benefits of polysaccharides from *Poria cocos* and their derivatives. Int. J. Biol. Macromol..

[37] Ríos J.-L. (2011). Chemical Constituents and Pharmacological Properties of *Poria cocos*. Planta Medica.

[38] Zhang Y., Huang J., Sun M., Duan Y., Wang L., Yu N., Peng D., Chen W., Wang Y. (2023). Preparation, characterization, antioxidant and antianemia activities of *Poria cocos* polysaccharide iron (III) complex. Heliyon.

[39] Tan Z., Zhang Q., Zhao R., Huang T., Tian Y., Lin Y. (2023). A Comparative Study on the Effects of Different Sources of Carboxymethyl Poria Polysaccharides on the Repair of DSS-Induced Colitis in Mice. Int. J. Mol. Sci..

[40] Lu M.-K., Chao C.-H., Hsu Y.-C. (2023). Advanced culture strategy shows varying bioactivities of sulfated polysaccharides of *Poria cocos*. Int. J. Biol. Macromol..

[41] Liu F., Zhang L., Feng X., Ibrahim S.A., Huang W., Liu Y. (2021). Immunomodulatory Activity of Carboxymethyl Pachymaran on Immunosuppressed Mice Induced by Cyclophosphamide. Molecules.

[42] Liu X., Wang X., Xu X., Zhang X. (2019). Purification, antitumor and anti-inflammation activities of an alkali-soluble and carboxymethyl polysaccharide CMP33 from *Poria cocos*. Int. J. Biol. Macromol..

[43] Cheng X., Cao L., Sun X., Zhou S., Zhu T., Zheng J., Liu S., Liu H. (2024). Metabolomic profile of plasma approach to investigate the mechanism of *Poria cocos* oligosaccharides attenuated LPS-induced acute lung injury in mice. J. Pharm. Biomed. Anal..

[44] Zhang D., Li H., Luo X., Liu D., Wei Q., Ye X. (2022). Integrated 16S rDNA, metabolomics, and TNF-α/NF-κB signaling pathway analyses to explain the modulatory effect of *Poria cocos* aqueous extract on anxiety-like behavior. Phytomedicine.

[45] Cox A.J., West N.P., Cripps A.W. (2015). Obesity, inflammation, and the gut microbiota. Lancet Diabetes Endocrinol..

[46] Teame T., Wang A., Xie M., Zhang Z., Yang Y., Ding Q., Gao C., Olsen R.E., Ran C., Zhou Z. (2020). Paraprobiotics and Postbiotics of Probiotic Lactobacilli, Their Positive Effects on the Host and Action Mechanisms: A Review. Front. Nutr..

[47] Moselhy S.N., Al-Nashwi A.A., Raya-Álvarez E., Abu Zaid F.O., Shalaby H.S.T., El-Khadragy M.F., Shahein M.R., Hafiz A.A., Aljehani A.A., Agil A. (2024). Physicochemical, microbiological, and sensory properties of healthy juices containing aloe vera gel and probiotics and their antidiabetic effects on albino rats. Front. Nutr..

[48] Li W., Yu J., Zhao J., Xiao X., Li W., Zang L., Yu J., Liu H., Niu X. (2021). *Poria cocos* polysaccharides reduces high-fat diet-induced arteriosclerosis in ApoE(-/-) mice by inhibiting inflammation. Phytother. Res..

[49] Wang J., Zheng D., Huang F., Zhao A., Kuang J., Ren Z., Chen T., Lei J., Lin J., Wang X. (2022). Theabrownin and *Poria cocos* Polysaccharide Improve Lipid Metabolism via Modulation of Bile Acid and Fatty Acid Metabolism. Front. Pharmacol..

[50] Zhu L., Chen G., Guo Y., Zheng J., Yang H., Sun X., Liu Y., Hu B., Liu H. (2022). Structural characterization of *Poria cocos* oligosaccharides and their effects on the hepatic metabolome in high-fat diet-fed mice. Food Funct..

[51] Zhao J., Niu X., Yu J., Xiao X., Li W., Zang L., Hu Z., Siu-Po Ip P., Li W. (2020). *Poria cocos* polysaccharides attenuated ox-LDL-induced inflammation and oxidative stress via ERK activated Nrf2/HO-1 signaling pathway and inhibited foam cell formation in VSMCs. Int. Immunopharmacol..

[52] Song D., Cao Z., Tickner J., Qiu H., Wang C., Chen K., Wang Z., Guo C., Dong S., Xu J. (2018). *Poria cocos* polysaccharide attenuates RANKL-induced osteoclastogenesis by suppressing NFATc1 activity and phosphorylation of ERK and STAT3. Arch. Biochem. Biophys..

[53] Wu Y., Li D., Wang H., Wan X. (2022). Protective Effect of *Poria cocos* Polysaccharides on Fecal Peritonitis-Induced Sepsis in Mice Through Inhibition of Oxidative Stress, Inflammation, Apoptosis, and Reduction of Treg Cells. Front. Microbiol..

[54] Jiang Y.-h., Wang L., Chen W.-d., Duan Y.-t., Sun M.-j., Huang J.-j., Peng D.-y., Yu N.-j., Wang Y.-y., Zhang Y. (2022). *Poria cocos* polysaccharide prevents alcohol-induced hepatic injury and inflammation by repressing oxidative stress and gut leakiness. Front. Nutr..

[55] Hu X., Hong B., Shan X., Cheng Y., Peng D., Hu R., Wang L., Chen W. (2023). The Effect of *Poria cocos* Polysaccharide PCP-1C on M1 Macrophage Polarization via the Notch Signaling Pathway. Molecules.

[56] Sun M., Yao L., Yu Q., Duan Y., Huang J., Lyu T., Yu N., Peng D., Chen W., Wang Y. (2024). Screening of *Poria cocos* polysaccharide with immunomodulatory activity and its activation effects on TLR4/MD2/NF-κB pathway. Int. J. Biol. Macromol..

[57] Tian H., Liu Z., Pu Y., Bao Y. (2019). Immunomodulatory effects exerted by *Poria cocos* polysaccharides via TLR4/TRAF6/NF-kappaB signaling in vitro and in vivo. Biomed. Pharmacother..

[58] He J., Lu J., Zhan L., Zheng D., Wang Y., Meng J., Li P., Zhao J., Zhang W. (2023). An Alkali-extracted polysaccharide from *Poria cocos* activates RAW264.7 macrophages via NF-κB signaling pathway. Arab. J. Chem..

[59] Lv Y., Yang Y., Chen Y., Wang D., Lei Y., Pan M., Wang Z., Xiao W., Dai Y. (2024). Structural characterization and immunomodulatory activity of a water-soluble polysaccharide from *Poria cocos*. Int. J. Biol. Macromol..

[60] Gu P., Xu P., Zhu Y., Zhao Q., Zhao X., Fan Y., Wang X., Ma N., Bao Y., Shi W. (2024). Structural characterization and adjuvant activity of a water soluble polysaccharide from *Poria cocos*. Int. J. Biol. Macromol..

[61] Shao H., Zhang C., Xiao N., Tan Z. (2020). Gut microbiota characteristics in mice with antibiotic-associated diarrhea. BMC Microbiol..

[62] Yu J., Chen Z., Zhou Q., Li P., Wu S., Zhou T., Gu Q. (2024). Exopolysaccharide from *Lacticaseibacillus paracasei* alleviates gastritis in Helicobacter pylori-infected mice by regulating gastric microbiota. Front. Nutr..

[63] Singh V.K., Hu X.-H., Singh A.K., Solanki M.K., Vijayaraghavan P., Srivastav R., Joshi N.K., Kumari M., Singh S.K., Wang Z. (2024). Precision nutrition-based strategy for management of human diseases and healthy aging: Current progress and challenges forward. Front. Nutr..

[64] Lu M.-K., Chao C.-H., Hsu Y.-C. (2021). Effect of carbohydrate-feeding strategy on the production, physiochemical changes, anti-inflammation activities of polysaccharides of *Poria cocos*. Int. J. Biol. Macromol..

[65] Xu D., Yuan L., Meng F., Lu D., Che M., Yang Y., Liu W., Nan Y. (2024). Research progress on antitumor effects of sea buckthorn, a traditional Chinese medicine homologous to food and medicine. Front. Nutr..

[66] Ma C., Lu J., Ren M., Wang Q., Li C., Xi X., Liu Z. (2023). Rapid identification of α-glucosidase inhibitors from Poria using spectrum-effect, component knock-out, and molecular docking technique. Front. Nutr..

[67] Mueed A., Shibli S., Al-Quwaie D.A., Ashkan M.F., Alharbi M., Alanazi H., Binothman N., Aljadani M., Majrashi K.A., Huwaikem M. (2023). Extraction, characterization of polyphenols from certain medicinal plants and evaluation of their antioxidant, antitumor, antidiabetic, antimicrobial properties, and potential use in human nutrition. Front. Nutr..

[68] Zhao H., Liu G., Li Y., Lu F., Yang N., Zhao J. (2024). Body fat ratio as a novel predictor of complications and survival after rectal cancer surgery. Front. Nutr..

[69] Ma Q., Ouyang Y., Meng F., Noolvi M.N., Avvaru S.P., More U.A., Aminabhavi T.M., Du M., Liu H., Zhuang Y. (2019). A review of pharmacological and clinical studies on the application of Shenling Baizhu San in treatment of Ulcerative colitis. J. Ethnopharmacol..

[70] Qin L., Huang D., Huang J., Qin F., Huang H. (2021). Integrated Analysis and Finding Reveal Anti–Liver Cancer Targets and Mechanisms of Pachyman (*Poria cocos* Polysaccharides). Front. Pharmacol..

[71] Zhu Q., Zhang P., Liu D., Tang L., Yu J., Zhang C., Jiang G. (2024). Glucosinolate extract from radish (*Raphanus sativus* L.) seed attenuates high-fat diet-induced obesity: Insights into gut microbiota and fecal metabolites. Front. Nutr..

[72] Li Y.-r., Liu S.-t., Gan Q., Zhang J., Chen N., Han C.-f., Geng W.-j., Wang B.-x., Han N., Jia S.-r. (2023). Four polysaccharides isolated from *Poria cocos* mycelium and fermentation broth supernatant possess different activities on regulating immune response. Int. J. Biol. Macromol..

[73] Yu Y., Shen M., Song Q., Xie J. (2018). Biological activities and pharmaceutical applications of polysaccharide from natural resources: A review. Carbohydr. Polym..

[74] Nowakowski P., Markiewicz-Zukowska R., Bielecka J., Mielcarek K., Grabia M., Socha K. (2021). Treasures from the forest: Evaluation of mushroom extracts as anti-cancer agents. Biomed. Pharmacother..

[75] Yang X., Lu S., Feng Y., Cao C., Zhang Y., Cheng S. (2023). Characteristics and properties of a polysaccharide isolated from *Wolfiporia cocos* as potential dietary supplement for IBS. Front. Nutr..

[76] Tang J., Nie J., Li D., Zhu W., Zhang S., Ma F., Sun Q., Song J., Zheng Y., Chen P. (2014). Characterization and antioxidant activities of degraded polysaccharides from *Poria cocos* sclerotium. Carbohydr. Polym..

[77] Koh A., De Vadder F., Kovatcheva-Datchary P., Bäckhed F. (2016). From Dietary Fiber to Host Physiology: Short-Chain Fatty Acids as Key Bacterial Metabolites. Cell.

[78] Pi X., Du Z., Teng W., Fu H., Hu L., Li J., Ding J., Yang X., Zhang Y. (2024). Characteristics of stachyose-induced effects on gut microbiota and microbial metabolites in vitro associated with obesity in children. Front. Nutr..

[79] Schell K.R., Fernandes K.E., Shanahan E., Wilson I., Blair S.E., Carter D.A., Cokcetin N.N. (2022). The Potential of Honey as a Prebiotic Food to Re-engineer the Gut Microbiome Toward a Healthy State. Front. Nutr..

[80] Zhang X.-Y., Chen J., Yi K., Peng L., Xie J., Gou X., Peng T., Tang L. (2020). Phlorizin ameliorates obesity-associated endotoxemia and insulin resistance in high-fat diet-fed mice by targeting the gut microbiota and intestinal barrier integrity. Gut Microbes.

[81] Zhu Z., Huang R., Huang A., Wang J., Liu W., Wu S., Chen M., Chen M., Xie Y., Jiao C. (2022). Polysaccharide from *Agrocybe cylindracea* prevents diet-induced obesity through inhibiting inflammation mediated by gut microbiota and associated metabolites. Int. J. Biol. Macromol..

[82] Manor O., Dai C.L., Kornilov S.A., Smith B., Price N.D., Lovejoy J.C., Gibbons S.M., Magis A.T. (2020). Health and disease markers correlate with gut microbiome composition across thousands of people. Nat. Commun..

[83] Li M., Yu L., Zhao J., Zhang H., Chen W., Zhai Q., Tian F. (2021). Role of dietary edible mushrooms in the modulation of gut microbiota. J. Funct. Foods.

[84] Song C., Huang F., Liu L., Zhou Q., Zhang D., Fang Q., Lei H., Niu H. (2022). Characterization and prebiotic properties of pectin polysaccharide from *Clausena lansium* (Lour.) Skeels fruit. Int. J. Biol. Macromol..

[85] Yang Y., Ye H., Zhao C., Ren L., Wang C., Georgiev M.I., Xiao J., Zhang T. (2021). Value added immunoregulatory polysaccharides of *Hericium erinaceus* and their effect on the gut microbiota. Carbohydr. Polym..

[86] Liu Z., Li L., Ma S., Ye J., Zhang H., Li Y., Sair A.T., Pan J., Liu X., Li X. (2020). High-Dietary Fiber Intake Alleviates Antenatal Obesity-Induced Postpartum Depression: Roles of Gut Microbiota and Microbial Metabolite Short-chain Fatty Acid Involved. J. Agric. Food Chem..

[87] Luo Y., Fang Q., Lai Y., Lei H., Zhang D., Niu H., Wang R., Song C. (2022). Polysaccharides from the leaves of *Polygonatum sibiricum* Red. regulate the gut microbiota and affect the production of short-chain fatty acids in mice. AMB Express.

[88] Khinsar K.H., Abdul S., Hussain A., Ud Din R., Lei L., Cao J., Abbasi M., Ur Rehman A., Farooqui N., Yi X. (2021). Anti-tumor effect of polysaccharide from *Pleurotus ostreatus* on H22 mouse Hepatoma ascites in-vivo and hepatocellular carcinoma in-vitro model. AMB Express.

[89] Do M.H., Lee H.B., Oh M.J., Jhun H., Choi S.Y., Park H.Y. (2021). Polysaccharide fraction from greens of *Raphanus sativus* alleviates high fat diet-induced obesity. Food Chem..

[90] Yu J., Xiang H., Xie Q. (2021). The difference of regulatory effect of two Inonotus obliquus extracts on high-fat diet mice in relation to the fatty acid elongation function of gut microbiota. Food Sci. Nutr..

[91] Wu J., Xu Y., Su J., Zhu B., Wang S., Liu K., Wang H., Shi S., Zhang Q., Qin L. (2020). Roles of gut microbiota and metabolites in a homogalacturonan-type pectic polysaccharide from *Ficus pumila* Linn. fruits mediated amelioration of obesity. Carbohydr. Polym..

[92] Yadav M.K., Kumari I., Singh B., Sharma K.K., Tiwari S.K. (2022). Probiotics, prebiotics and synbiotics: Safe options for next-generation therapeutics. Appl. Microbiol. Biotechnol..

[93] Schroeder B.O., Backhed F. (2016). Signals from the gut microbiota to distant organs in physiology and disease. Nat. Med..

[94] Yi Y., Xu W., Wang H.X., Huang F., Wang L.M. (2020). Natural polysaccharides experience physiochemical and functional changes during preparation: A review. Carbohydr. Polym..

[95] Feng W., Ao H., Peng C. (2018). Gut Microbiota, Short-Chain Fatty Acids, and Herbal Medicines. Front. Pharmacol..

[96] Xue H., Mei C.F., Wang F.Y., Tang X.D. (2023). Relationship among Chinese herb polysaccharide (CHP), gut microbiota, and chronic diarrhea and impact of CHP on chronic diarrhea. Food Sci. Nutr..

[97] He Y., Fu L., Li Y., Wang W., Gong M., Zhang J., Dong X., Huang J., Wang Q., Mackay C.R. (2021). Gut microbial metabolites facilitate anticancer therapy efficacy by modulating cytotoxic CD8+ T cell immunity. Cell Metab..

[98] Zhang D., Jian Y.-P., Zhang Y.-N., Li Y., Gu L.-T., Sun H.-H., Liu M.-D., Zhou H.-L., Wang Y.-S., Xu Z.-X. (2023). Short-chain fatty acids in diseases. Cell Commun. Signal..

[99] Coutzac C., Jouniaux J.-M., Paci A., Schmidt J., Mallardo D., Seck A., Asvatourian V., Cassard L., Saulnier P., Lacroix L. (2020). Systemic short chain fatty acids limit antitumor effect of CTLA-4 blockade in hosts with cancer. Nat. Commun..

[100] Wang Z., Li Y., Liao W., Huang J., Liu Y., Li Z., Tang J. (2022). Gut microbiota remodeling: A promising therapeutic strategy to confront hyperuricemia and gout. Front. Cell. Infect. Microbiol..

[101] Brown E.M., Clardy J., Xavier R.J. (2023). Gut microbiome lipid metabolism and its impact on host physiology. Cell Host Microbe.

[102] Kindt A., Liebisch G., Clavel T., Haller D., Hormannsperger G., Yoon H., Kolmeder D., Sigruener A., Krautbauer S., Seeliger C. (2018). The gut microbiota promotes hepatic fatty acid desaturation and elongation in mice. Nat. Commun..

[103] de Vos W.M., Tilg H., Van Hul M., Cani P.D. (2022). Gut microbiome and health: Mechanistic insights. Gut.

[104] Xie Y.-K., Pan X.-Y., Liang X.-R., Zhai K.-F., Yu Q. (2025). Research progress on structural characterization and bioactivities of *Poria cocos* and *Ganoderma* polysaccharides. Food Med. Homol..

[105] Sun Y., Liu Z., Pi Z., Song F., Wu J., Liu S. (2021). *Poria cocos* could ameliorate cognitive dysfunction in APP/PS1 mice by restoring imbalance of Aβ production and clearance and gut microbiota dysbiosis. Phytother. Res..

[106] Chen P., Chen X., Hao L., Du P., Li C., Han H., Xu H., Liu L. (2021). The bioavailability of soybean polysaccharides and their metabolites on gut microbiota in the simulator of the human intestinal microbial ecosystem (SHIME). Food Chem..

[107] Yu J., Xiang J.Y., Xiang H., Xie Q. (2020). Cecal Butyrate (Not Propionate) Was Connected with Metabolism-Related Chemicals of Mice, Based on the Different Effects of the Two Inonotus obliquus Extracts on Obesity and Their Mechanisms. ACS Omega.

[108] Wang Y.-Z., Zhang J., Zhao Y.-L., Li T., Shen T., Li J.-Q., Li W.-Y., Liu H.-G. (2013). Mycology, cultivation, traditional uses, phytochemistry and pharmacology of *Wolfiporia cocos* (Schwein.) Ryvarden et Gilb.: A review. J. Ethnopharmacol..

[109] Sun B., Huang B., Sica V.P., Baker T.R., Pfuhler S. (2021). A genotoxicity assessment approach for botanical materials demonstrated with *Poria cocos*. Food Chem. Toxicol..

[110] Khan I., Huang G., Li X., Leong W., Xia W., Hsiao W.L.W. (2018). Mushroom polysaccharides from Ganoderma lucidum and *Poria cocos* reveal prebiotic functions. J. Funct. Foods.

[111] Duan Y., Huang J., Sun M., Jiang Y., Wang S., Wang L., Yu N., Peng D., Wang Y., Chen W. (2023). *Poria cocos* polysaccharide improves intestinal barrier function and maintains intestinal homeostasis in mice. Int. J. Biol. Macromol..

[112] Wang M., Xie Z., Li L., Chen Y., Li Y., Wang Y., Lu B., Zhang S., Ma F., Ma C. (2019). Supplementation with compound polysaccharides contributes to the development and metabolic activity of young rat intestinal microbiota. Food Funct..

[113] Lai Y., Yu H., Deng H., Fang Q., Lei H., Liu L., Wu N., Guo X., Song C. (2022). Three main metabolites from *Wolfiporia cocos* (F. A. *Wolf*) Ryvarden & Gilb regulate the gut microbiota in mice: A comparative study using microbiome-metabolomics. Front. Pharmacol..

[114] Lan K., Yang H., Zheng J., Hu H., Zhu T., Zou X., Hu B., Liu H. (2023). *Poria cocos* oligosaccharides ameliorate dextran sodium sulfate-induced colitis mice by regulating gut microbiota dysbiosis. Food Funct..

[115] Song X., Wang W., Liu L., Zhao Z., Shen X., Zhou L., Zhang Y., Peng D., Nian S. (2024). *Poria cocos* Attenuated DSS-Induced Ulcerative Colitis via NF-κB Signaling Pathway and Regulating Gut Microbiota. Molecules.

[116] Yu J., Hu Q., Liu J., Luo J., Liu L., Peng X. (2022). Metabolites of gut microbiota fermenting *Poria cocos* polysaccharide alleviates chronic nonbacterial prostatitis in rats. Int. J. Biol. Macromol..

[117] Liu J., Yu J., Peng X. (2020). *Poria cocos* Polysaccharides Alleviates Chronic Nonbacterial Prostatitis by Preventing Oxidative Stress, Regulating Hormone Production, Modifying Gut Microbiota, and Remodeling the DNA Methylome. J. Agric. Food Chem..

[118] Liu J., Liu L., Zhang G., Peng X. (2021). *Poria cocos* polysaccharides attenuate chronic nonbacterial prostatitis by targeting the gut microbiota: Comparative study of *Poria cocos* polysaccharides and finasteride in treating chronic prostatitis. Int. J. Biol. Macromol..

[119] Sun S.-S., Wang K., Ma K., Bao L., Liu H.-W. (2019). An insoluble polysaccharide from the sclerotium of *Poria cocos* improves hyperglycemia, hyperlipidemia and hepatic steatosis in ob/ob mice via modulation of gut microbiota. Chin. J. Nat. Med..

[120] Jiang S., Liang C., Wan X., Po Lai K., Li R., Chen J., Liu J. (2024). Integrative analysis reveals the anti-obesity roles of *Poria cocos* polysaccharides through beneficial effects on gut microbiota. J. Funct. Foods.

[121] Zhu L., Ye C., Hu B., Xia H., Bian Q., Liu Y., Kong M., Zhou S., Liu H. (2022). Regulation of gut microbiota and intestinal metabolites by *Poria cocos* oligosaccharides improves glycolipid metabolism disturbance in high-fat diet-fed mice. J. Nutr. Biochem..

[122] Tan Y.-y., Yue S.-r., Lu A.-p., Zhang L., Ji G., Liu B.-c., Wang R.-r. (2022). The improvement of nonalcoholic steatohepatitis by *Poria cocos* polysaccharides associated with gut microbiota and NF-κB/CCL3/CCR1 axis. Phytomedicine.

[123] Sun S., Wang K., Sun L., Cheng B., Qiao S., Dai H., Shi W., Ma J., Liu H. (2020). Therapeutic manipulation of gut microbiota by polysaccharides of *Wolfiporia cocos* reveals the contribution of the gut fungi-induced PGE2 to alcoholic hepatic steatosis. Gut Microbes.

[124] Zhang D.-d., Li H.-j., Zhang H.-r., Ye X.-c. (2022). *Poria cocos* water-soluble polysaccharide modulates anxiety-like behavior induced by sleep deprivation by regulating the gut dysbiosis, metabolic disorders and TNF-α/NF-κB signaling pathway. Food Funct..

[125] Ye C., Gao Z.-H., Chen K.-Q., Lu F.-G., Wei K. (2023). Research on Pachymaran to Ameliorate CsA-Induced Immunosuppressive Lung Injury by Regulating Microflora Metabolism. Microorganisms.

[126] Yin L., Huang G., Khan I., Su L., Xia W., Law B.Y.K., Wong V.K.W., Wu Q., Wang J., Leong W.K. (2022). *Poria cocos* polysaccharides exert prebiotic function to attenuate the adverse effects and improve the therapeutic outcome of 5-FU in ApcMin/+ mice. Chin. Med..

[127] Zou Y.T., Zhou J., Wu C.Y., Zhang W., Shen H., Xu J.D., Zhang Y.Q., Long F., Li S.L. (2021). Protective effects of *Poria cocos* and its components against cisplatin-induced intestinal injury. J. Ethnopharmacol..

[128] Xu H., Wang S., Jiang Y., Wu J., Chen L., Ding Y., Zhou Y., Deng L., Chen X. (2023). *Poria cocos* Polysaccharide Ameliorated Antibiotic-Associated Diarrhea in Mice via Regulating the Homeostasis of the Gut Microbiota and Intestinal Mucosal Barrier. Int. J. Mol. Sci..

[129] Lai Y., Deng H., Fang Q., Ma L., Lei H., Guo X., Chen Y., Song C. (2023). Water-Insoluble Polysaccharide Extracted from *Poria cocos* Alleviates Antibiotic-Associated Diarrhea Based on Regulating the Gut Microbiota in Mice. Foods.

[130] Mekonnen S.A., Merenstein D., Fraser C.M., Marco M.L. (2020). Molecular mechanisms of probiotic prevention of antibiotic-associated diarrhea. Curr. Opin. Biotechnol..

[131] Chang C.J., Lin C.S., Lu C.C., Martel J., Ko Y.F., Ojcius D.M., Tseng S.F., Wu T.R., Chen Y.Y., Young J.D. (2015). Ganoderma lucidum reduces obesity in mice by modulating the composition of the gut microbiota. Nat. Commun..

[132] Zhang J., Shu D., Cheng X., Tian T., Xiao K., Zhang D., Yang J. (2023). Effect of plant polysaccharides (*Poria cocos* and *Astragalus* polysaccharides) on immune responses and intestinal microbiota of Dabry’s sturgeons. Biosci. Microbiota Food Health.

[133] Vinolo M.A.R., Rodrigues H.G., Nachbar R.T., Curi R. (2011). Regulation of Inflammation by Short Chain Fatty Acids. Nutrients.

[134] Pickard J.M., Zeng M.Y., Caruso R., Nunez G. (2017). Gut microbiota: Role in pathogen colonization, immune responses, and inflammatory disease. Immunol. Rev..

[135] Wang L., Wang S., Zhang Q., He C., Fu C., Wei Q. (2022). The role of the gut microbiota in health and cardiovascular diseases. Mol. Biomed..

[136] Wang Y., Tang B., Long L., Luo P., Xiang W., Li X., Wang H., Jiang Q., Tan X., Luo S. (2021). Improvement of obesity-associated disorders by a small-molecule drug targeting mitochondria of adipose tissue macrophages. Nat. Commun..

[137] Peng X., Jia C., Chi H., Wang P., Fu H., Li Y., Wang Q. (2022). Efficacy and Pharmacological Mechanism of *Poria cocos*-Based Formulas Combined with Chemotherapy for Ovarian Cancer: A Integrated Systems Pharmacology Study. Front. Pharmacol..

[138] Willing B.P., Russell S.L., Finlay B.B. (2011). Shifting the balance: Antibiotic effects on host–microbiota mutualism. Nat. Rev. Microbiol..

[139] Kopacz K., Phadtare S. (2022). Probiotics for the Prevention of Antibiotic-Associated Diarrhea. Healthcare.

[140] Tanır Basaranoğlu S., Karaaslan A., Salı E., Çiftçi E., Gayretli Aydın Z.G., Aldemir Kocabaş B., Kaya C., Şen Bayturan S., Kara S.S., Yılmaz Çiftdoğan D. (2023). Antibiotic associated diarrhea in outpatient pediatric antibiotic therapy. BMC Pediatr..

[141] Ji X., Guo J., Cao T., Zhang T., Liu Y., Yan Y. (2023). Review on mechanisms and structure-activity relationship of hypoglycemic effects of polysaccharides from natural resources. Food Sci. Hum. Wellness.

[142] Hou C., Yin M., Lan P., Wang H., Nie H., Ji X. (2021). Recent progress in the research of *Angelica sinensis* (Oliv.) Diels polysaccharides: Extraction, purification, structure and bioactivities. Chem. Biol. Technol. Agric..

[143] Ji X., Hou C., Gao Y., Xue Y., Yan Y., Guo X. (2020). Metagenomic analysis of gut microbiota modulatory effects of jujube (*Ziziphus jujuba* Mill.) polysaccharides in a colorectal cancer mouse model. Food Funct..

[144] Wang Y., Yu Y., Mao J. (2009). Carboxymethylated β-Glucan Derived from *Poria cocos* with Biological Activities. J. Agric. Food Chem..

[145] Zhao M., Guan Z., Tang N., Cheng Y. (2023). The differences between the water- and alkaline-soluble *Poria cocos* polysaccharide: A review. Int. J. Biol. Macromol..

[146] Li X., Ma L., Zhang L. (2019). Molecular basis for *Poria cocos* mushroom polysaccharide used as an antitumor drug in China. Prog. Mol. Biol. Transl. Sci..

[147] Yu C., Dong Q., Chen M., Zhao R., Zha L., Zhao Y., Zhang M., Zhang B., Ma A. (2023). The Effect of Mushroom Dietary Fiber on the Gut Microbiota and Related Health Benefits: A Review. J. Fungi.

[148] Qiu Y., Lin G., Liu W., Zhang F., Linhardt R.J., Wang X., Zhang A. (2024). Bioactive compounds in *Hericium erinaceus* and their biological properties: A review. Food Sci. Hum. Wellness.

[149] Wu P., Tan H., Zhan J., Wang W., Hu T., Li S. (2020). Optimization of Bioprocess Extraction of *Poria cocos* Polysaccharide (PCP) with Aspergillus niger β-Glucanase and the Evaluation of PCP Antioxidant Property. Molecules.

